# Socioeconomic Differences in Adolescent Health-Related Behavior Differ by Gender

**DOI:** 10.2188/jea.JE20120133

**Published:** 2013-05-05

**Authors:** Lukas Pitel, Andrea Madarasová Gecková, Sijmen A. Reijneveld, Jitse P. van Dijk

**Affiliations:** 1 Institute of Experimental Psychology, Slovak Academy of Sciences, Bratislava, Slovakia; 2 Graduate School Kosice Institute for Society and Health, Medical Faculty, PJ Safarik University, Kosice, Slovakia; 3 Public Health Institute, Department of Health Psychology, Medical Faculty, PJ Safarik University, Kosice, Slovakia; 4 Department of Social Medicine, University Medical Centre Groningen, University of Groningen, Groningen, The Netherlands

**Keywords:** gender differences, socioeconomic differences, adolescents, health-related behavior

## Abstract

**Background:**

Many studies of adolescent health-related behaviors have assessed the effects of gender and parental socioeconomic position (SEP) but not their mutual modification. We investigated socioeconomic differences in health-related behaviors among Slovak adolescents and the potential modification of those differences by gender.

**Methods:**

Data were collected in 2006 (*n* = 3547; 49.4% boys; mean [SD] age, 14.3 [0.6] years; response rate, 93.5%). The sample comprised students in the eighth and ninth grades of randomly selected elementary schools in Slovakia. Gender-specific prevalence rates for 9 types of health-related behaviors, including nutritional behavior, physical activity and substance use, were calculated for 3 socioeconomic groups, which were defined by the highest educational level attained by both parents. Gender differences in socioeconomic gradients for health-related behaviors were tested.

**Results:**

Socioeconomic differences were found in nutritional behavior, physical activity, and smoking. Adolescents with lower parental education behaved less healthily. The largest relative socioeconomic difference was no daily vegetable consumption among girls (90.3% of those with high SEP vs 95.2% of those with middle SEP; odds ratio, 2.33). Regarding no daily fruit consumption, differences among girls were 1.51 times and 1.92 times as large as those among boys for children with medium and low SEP, respectively, as compared with those with high SEP.

**Conclusions:**

Socioeconomic differences in health-related behavior were small, especially for nutritional behavior and physical activity. Interventions that aim to improve health-related behaviors among adolescents with lower SEP should focus on these 2 behaviors, particularly on healthy nutrition in girls with low SEP.

## INTRODUCTION

Health-related behavior (HRB) has traditionally been defined as any kind of behavior undertaken by individuals that potentially influences their health.^1^ Because adolescence is a crucial period of development with respect to future HRB habits,^2^ it has implications for health and illness later in life. In childhood, HRB is strongly subject to parental influences: parents set the standards for the behaviors of their children and control their compliance. With the beginning of adolescence, young people increasingly decide their own behavior and spend progressively more time with their peers, which may partially explain why adolescence is the key period for initiation of substance use.^3^

However, particular behaviors may be initiated and consolidated at different stages of childhood and adolescence. Dietary and exercise habits, although established more permanently during adolescence, often originate in childhood.^4^ Nutritional behavior seems to be influenced more by parents than by peers^5^ and is partially associated with parental socioeconomic position (SEP).^6^^–^^8^ Substance use is mostly initiated during adolescence^9^^,^^10^ and seems to be influenced to a greater degree by friends and classmates.^5^^,^^11^ This may explain why some studies have found socioeconomic (SE) differences in adolescent HRB, while others have not.^6^^,^^12^^,^^13^ When SE differences were found, they were less pronounced than in childhood or adulthood.^6^^,^^14^

Another important predictor of adolescent and adult health behavior is gender.^15^ In general, boys cope with developmental tasks and transitions in a more externalizing way, eg, by physical activity but also through substance use. Girls tend to cope with these tasks in a more internalizing way, eg, by problematic nutritional behaviors.^15^ Therefore their substance use prevalence is usually lower than that of boys.^16^^,^^17^ However, in many countries there has been a trend in recent decades toward equalization or even reversal of these gender differences.^18^^–^^22^ Richter^3^ hypothesizes that this gender convergence in substance use concerns less risky behavioral patterns only. As for nutritional behavior, girls usually consume more fruit and fewer soft drinks,^22^ but also more frequently skip meals^22^ and are more often dissatisfied with their bodies, than boys.^23^

Due to differences in socialization patterns, it can be assumed that there are gender differences in the association between adolescent HRB and parental characteristics such as parental SEP. Most studies in this field now assess the effects of gender and parental SEP but not their joint effects.^5^^,^^16^ Few studies have assessed the SE gradient in HRB separately in boys and girls. Furthermore, only a small number of studies have examined a range of health behaviors instead of focusing on a single behavior.^3^

There are even fewer studies from the former communist countries in Central and Eastern Europe on the health behaviors of younger age groups and SE differences in these behaviors. The Health Behavior in School-aged Children (HBSC) report, published in 2008,^22^ revealed that SE gradient, measured by Family Affluence Scale, in Central and Eastern Europe countries was similar to that in the rest of Europe for most health behaviors. The exception was breakfast consumption, which—unlike in the rest of Europe—had mostly no traditional gradient in Central and Eastern Europe. This was true for both genders. However, affluence is only 1 possible measure of SEP (along with income, occupation, and education), and each of these indicators may be associated with adolescent HRB in a different way.

We assessed SE differences, as measured by parental education, in HRB among Slovak adolescents in relation to nutritional behavior, physical inactivity, smoking, frequent alcohol consumption, drunkenness, and cannabis use, as well as the potential modification of these differences by gender.

## METHODS

### Subjects

The sample consisted of 3725 adolescents in the eighth and ninth grades of ordinary elementary schools across Slovakia. Data were collected from October through December 2006 in the major cities of Bratislava (450 000 inhabitants, Western Slovakia), Kosice (240 000 inhabitants, Eastern Slovakia), and Zilina (85 000 inhabitants, Central Slovakia), and from several other towns and villages, mostly in Eastern Slovakia, with a population less than 40 000. The Slovak Institute of Information and Prognosis of Education provided us with a list of all elementary schools in these municipalities. From this list, we randomly selected individual schools until we had a sufficient number of participants. The schools were contacted and asked to participate. Only 1 declined to do so. Parents were contacted by school administrators before the study and were given the opportunity to opt out if they disagreed with their child’s participation. Schools that had agreed to participate were visited, and data were collected from among all students in the eighth and ninth grades, unless their parents had requested to opt out. We excluded 178 cases from special schools attended by students with special educational needs. Ultimately, data from a sample consisting of 3547 adolescents (mean [SD] age, 14.3 [0.6] years; 49.4% boys; response rate, 93.5%) were analyzed. The primary reasons for nonresponse were illness and other types of absence. The study was approved by the Ethics Committee of the Faculty of Science at P.J. Safarik University in Kosice.

### Instruments

*Sociodemographic measures* included gender, age, and highest educational level of the father and mother, categorized as 3 SEP levels: low (elementary school and apprenticeship), middle (completion of secondary school, including graduation examinations), and high (university education). The SEP level of the adolescent was defined as the highest educational level attained by both parents, as in previous studies.^14^^,^^24^^–^^27^

*Health-related behavior* concerned frequency of having breakfast, consumption of fruit, vegetables, and sweets, physical activity, smoking, and frequency of alcohol consumption, drunkenness, and cannabis use. The wording of the questions was derived from the questions used in the HBSC studies,^19^ and the answers were dichotomized.

*Having breakfast* was assessed by the question: “How many times a week do you have breakfast?” Respondents could choose from the following options: not even once, 1 to 2 times, 3 to 4 times, 5 to 6 times, and every day. All those who selected any option other than “every day” were labeled as behaving unhealthily.

*Consumption of fruit* was measured by the question: “How many times a week do you eat fresh fruit?” Respondents could choose from the following options: not even once, 1 to 2 times, 3 to 4 times, 5 to 6 times, every day. All those who selected any option other than “every day” were labeled as behaving unhealthily. Similar wording, the same options, and the same cut-off point were used to assess *consumption of vegetables*.

For *consumption of sweets* (without further specification), similar wording and the same options were used. All those who reported daily consumption of sweets were considered to be behaving unhealthily.

*Physical inactivity* was measured by the question: “How many days per week are you usually physically active for more than 60 minutes?” Respondents could choose the number of days, from 0 to 7. Those who were physically active fewer than 5 days per week were considered to be behaving unhealthily.

*Cigarette smoking* was measured by the question: “Have you ever smoked a cigarette (even just once)?” Respondents could choose from the following options: “I do not smoke”, “I have already tried smoking”, “I used to smoke but I have stopped completely”, “I smoke occasionally but not daily”, and “I smoke daily”. Unhealthy behavior was defined as smoking daily or occasionally.

*Drunkenness* was measured by the question: “Have you been drunk during the past 4 weeks?” In everyday Slovak language, the word “drunk” refers only to drunkenness induced by a high dose of alcohol. Respondents could choose from the following options: not even once, 1 to 2 times, or 3 or more times. All subjects who reported having been drunk at least once in the previous 4 weeks were labeled as behaving unhealthily.

*Alcohol consumption* was measured by the question: “How many times during the past 4 weeks have you drunk alcohol (≥1 glass of beer, brandy, or wine)?” Respondents could choose from the following options: not even once, 1 to 2 times, or 3 or more times. All subjects who reported having drunk alcohol 3 or more times during the previous 4 weeks were labeled as behaving unhealthily.

*Cannabis use* was measured by the question: “Have you ever smoked hashish or marijuana?” Respondents could choose from the following options: “No, never”, “I have tried it already”, “I smoke from time to time but not daily”, or “I smoke daily”. All those who reported having smoked hashish or marijuana daily or from time to time were labeled as behaving unhealthily.

The dichotomizations were established in accordance with previous research,^28^^–^^31^ so as to differentiate behaviors with potential health consequences, discriminate the population at risk in a suitable way, and enable comparisons with previous, similar studies.^28^^–^^31^

### Procedure

The questionnaire was completed in respondents’ classrooms, in the absence of teachers, under the guidance of field workers and on a voluntary and anonymous basis. The study was approved by the local ethics committee.

### Data analysis

Prevalence rates for the 9 HRB categories both overall and for each SE group were computed and analyzed separately by gender. Next, age-adjusted odds ratios (ORs) and 95% CIs for the medium and low SE groups, as compared with the high SE group, were computed for both genders. Additionally, the interactions of the effects of gender and SEP on all separate HRBs for the 3 SEP levels were analyzed using a logistic regression model, also adjusted for age. Finally, indices of dissimilarity (IDs) for each type of HRB were calculated for boys and girls. The index of dissimilarity represents the percentage of all cases (individuals) that must be redistributed to obtain the same prevalence rate in all SE groups.^32^ The statistical analyses were performed using SPSS 16.0.

To account for clustering of student outcomes per class, the logistic regression analyses were repeated on 2006 data using MLWiN 2.02 (http://www.cmm.bristol.ac.uk/MLwiN/).

## RESULTS

The sample comprised 1705 boys and 1749 girls: 1342 (39.8%) had parents with a university degree, 1650 (48.9%) had parents who were high school graduates, and 379 (11.2%) had parents with a lower educational degree.

A power analysis was not performed ex ante. Post-hoc power analyses were performed on gender differences in prevalence rates and on SE gradients per gender,^33^ both at *P* less than 0.05, using PASS. Regarding gender differences, the power to detect differences in prevalence rates of smoking as found (ie, 2.7%) was about 45%. The power was much higher for HRBs with larger differences, like alcohol use, cannabis use, physical activity, and daily breakfast. Regarding socioeconomic gradients in smoking, the power was 58% for girls and about 28% for boys. The power to detect gradients varied according to the strength of the gradient and was lowest for alcohol use in girls (4%) and highest for no daily fruit consumption in girls (99.8%).

Multilevel analyses showed very limited clustering by class and yielded identical or nearly identical ORs and 95% CIs,^18^^,^^34^ eg, the ORs (95% CI) for males versus females regarding alcohol use, smoking, and lack of physical activity were 1.26 (0.89–1.80), 0.80 (0.59–1.08), and 0.58 (0.43–0.78), respectively in the multilevel analyses, as compared with 1.27 (0.89–1.80), 0.79 (0.59–1.06), and 0.58 (0.44–0.78), respectively, in ordinary logistic regression. Therefore, all analyses were performed using ordinary logistic regressions with SPSS version 16.0.

The results of these analyses are presented in Tables 1, 2, and 3. The prevalence of unhealthy behaviors varied widely: from 3.4% for cannabis use (among girls) to 93.2% for no daily vegetables (among girls). Prevalence rates for frequent alcohol consumption and daily and occasional cannabis use were significantly higher among boys than among girls. The prevalence rates for skipping breakfast, physical inactivity, and smoking were significantly higher among girls than among boys (Table 1).

**Table 1.** Overall prevalence rates for health-related behaviors by gender, and age-adjusted ORs and 95% CIs for males versus females

Behavior	Boys		Girls		OR (95% CI)	*P*
	
	*n*	in %	*n*	in %		
No daily breakfast	678/1514	44.8	923/1605	57.5	0.59 (0.50–0.69)	<0.001
No daily fruit	1044/1514	69.0	1115/1603	69.6	1.04 (0.87–1.24)	0.703
No daily vegetables	1381/1495	92.4	1480/1588	93.2	1.19 (0.87–1.64)	0.282
Daily sweets	578/1510	38.3	633/1591	39.8	0.88 (0.74–1.03)	0.105
Physical activity <5 times/week	952/1575	60.4	1295/1642	78.9	0.41 (0.34–0.48)	<0.001
Smoking (daily or occasionally)	337/1619	20.8	394/1679	23.5	0.73 (0.58–0.91)	0.005
Drunk at least once in last 4 weeks	310/1584	19.6	295/1662	17.7	0.90 (0.71–1.16)	0.417
Alcohol ≥3 times in last 4 weeks	281/1608	17.5	207/1669	12.4	1.56 (1.21–2.00)	0.001
Cannabis (daily or occasionally)	119/1605	7.4	57/1669	3.4	2.77 (1.86–4.12)	<0.001

**Table 2.** Prevalence rates for nutritional behavior, physical inactivity, and several types of substance use; age-adjusted ORs and 95% CIs by SEP; and indices of dissimilarity, expressed as percentages (ID)

	Boys					Girls				
	
	*n*	in %	ID (%)	OR (95% CI)	*P*	*n*	in %	ID (%)	OR (95% CI)	*P*
No daily breakfast			2.70		0.085			2.97		0.038
High SEP	284/675	42.1		1		305/578	52.8		1	
Medium SEP	325/707	46.0		1.16 (0.94–1.45)		485/821	59.1		1.22 (0.98–1.52)	
Low SEP	69/132	52.3		1.51 (1.02–2.22)		133/206	64.6		1.50 (1.07–2.09)	
No daily fruit			0.42		0.912			3.68		<0.001
High SEP	459/672	68.3		1		361/578	62.5		1	
Medium SEP	491/710	69.2		1.02 (0.80–1.28)		591/819	72.2		1.53 (1.21–1.93)	
Low SEP	94/132	71.2		1.10 (0.72–1.68)		163/206	79.1		2.05 (1.40–3.00)	
No daily vegetables			0.50		0.326			1.14		0.001
High SEP	612/670	91.3		1		519/575	90.3		1	
Medium SEP	644/693	92.9		1.25 (0.83–1.89)		771/810	95.2		2.33 (1.51–3.60)	
Low SEP	125/132	94.7		1.76 (0.74–4.19)		190/203	93.6		1.63 (0.87–3.06)	
Daily sweets			1.98		0.070			1.04		0.802
High SEP	248/673	36.8		1		223/577	38.6		1	
Medium SEP	268/705	38.0		1.07 (0.86–1.35)		327/812	40.3		1.08 (0.86–1.34)	
Low SEP	62/132	47.0		1.58 (1.07–2.33)		83/202	41.1		1.07 (0.77–1.49)	
Physical activity <5 times/week			1.76		0.049			1.45		0.045
High SEP	407/701	58.1		1		441/583	75.6		1	
Medium SEP	453/738	61.4		1.19 (0.96–1.48)		666/835	79.8		1.25 (0.97–1.62)	
Low SEP	92/136	67.6		1.60 (1.06–2.40)		188/224	83.9		1.62 (1.07–2.45)	
Smoking (daily or occasionally)			3.40		0.515			5.04		0.054
High SEP	138/718	19.2		1		120/596	20.1		1	
Medium SEP	166/758	21.9		1.17 (0.90–1.52)		215/855	25.1		1.38 (1.06–1.78)	
Low SEP	33/143	23.1		1.09 (0.69–1.72)		59/228	25.9		1.27 (0.88–1.84)	
Drunk at least once in last 4 weeks			3.09		0.445			2.74		0.657
High SEP	128/703	18.2		1		97/592	16.4		1	
Medium SEP	150/745	20.1		1.18 (0.90–1.55)		154/844	18.2		1.14 (0.86–1.52)	
Low SEP	32/136	23.5		1.20 (0.75–1.91)		44/226	19.5		1.13 (0.75–1.69)	
Alcohol ≥3 times in last 4 weeks			3.06		0.372			0.64		0.798
High SEP	116/713	16.3		1		73/591	12.4		1	
Medium SEP	134/752	17.8		1.17 (0.88–1.55)		107/852	12.6		0.98 (0.71–1.35)	
Low SEP	31/143	21.7		1.34 (0.84–2.13)		27/226	11.9		0.85 (0.53–1.38)	
Cannabis (daily or occasionally)			6.48		0.372			3.13		0.464
High SEP	45/711	6.3		1		21/593	3.5		1	
Medium SEP	62/751	8.3		1.34 (0.89–2.01)		30/846	3.5		1.02 (0.58–1.83)	
Low SEP	12/143	8.5		1.18 (0.59–2.24)		6/226	2.7		0.56 (0.21–1.53)	

**Table 3.** Gender modification of associations of SEP with health-related behaviors, adjusted for age: odds ratios (95% CI)

	No daily breakfast	No daily fruit	No daily vegetables	Daily sweets	Physical activity <5 times/wk	Smoking (daily or occasionally)	Drunk at least once in last 4 weeks	Alcohol ≥3 times in last 4 weeks	Cannabis (daily or occasionally)
Model with main effects only									
Age	1.14 (1.01–1.28)*	1.26 (1.11–1.44)*	1.00 (0.80–1.26)	1.06 (0.94–1.20)	1.10 (0.97–1.25)	1.51 (1.31–1.73)***	1.66 (1.44–1.93)***	1.49 (1.27–1.75)***	1.64 (1.28–2.11)***
Gender									
Male	1	1	1	1	1	1	1	1	1
Female	1.66 (1.43–1.92)***	1.05 (0.90–1.23)	1.04 (0.78–1.38)	1.07 (0.03–1.25)	2.35 (2.00–2.77)***	1.18 (0.99–1.40)	0.91 (0.76–1.10)	0.69 (0.56–0.84)***	0.45 (0.32–0.62)***
SEP									
High	1**	1**	1**	1	1**	1*	1	1	1
Medium	1.20 (1.02–1.39)*	1.25 (1.06–1.47)**	1.69 (1.25–2.29)**	1.08 (0.92–1.26)	1.22 (1.03–1.44)*	1.27 (1.05–1.52)*	1.16 (0.95–1.41)	1.08 (0.88–1.34)	1.23 (0.88–1.72)
Low	1.50 (1.16–1.93)**	1.55 (1.17–2.05)**	1.60 (0.96–2.65)	1.25 (0.97–1.61)	1.61 (1.20–2.15)**	1.18 (0.89–1.57)	1.15 (0.85–1.56)	1.07 (0.77–1.49)	0.91 (0.51–1.61)

Model with main effects and the interaction terms									
Age	1.13 (1.01–1.28)*	1.26 (1.11–1.43)***	1.00 (0.79–1.25)	1.06 (0.94–1.20)	1.10 (0.97–1.25)	1.51 (1.31–1.73)***	1.66 (1.44–1.93)***	1.50 (1.27–1.75)***	1.65 (1.28–2.11)***
Gender									
Male	1	1	1	1	1	1	1	1	1
Female	1.61 (1.28–2.03)***	0.81 (0.64–1.03)	0.82 (0.55–1.22)	1.12 (0.88–1.41)	2.29 (1.79–2.93)***	1.07 (0.80–1.41)	0.94 (0.70–1.27)	0.80 (0.58–1.10)	0.56 (0.32–0.96)*
SEP									
High	1	1	1	1	1	1	1	1	1
Medium	1.16 (0.93–1.45)	1.01 (0.80–1.28)	1.25 (0.83–1.90)	1.07 (0.86–1.34)	1.19 (0.96–1.48)	1.17 (0.90–1.51)	1.18 (0.90–1.55)	1.17 (0.88–1.55)	1.34 (0.89–2.01)
Low	1.49 (1.01–2.19)*	1.08 (0.71–1.66)	1.80 (0.75–4.28)	1.57 (1.07–2.32)*	1.59 (1.06–2.40)*	1.10 (0.70–1.74)	1.21 (0.76–1.93)	1.34 (0.84–2.14)	1.18 (0.59–2.36)
Female (vs male) gender by SEP									
High	1	1*	1	1	1	1	1	1	1
Medium	1.06 (0.77–1.44)	1.51 (1.09–2.10)*	1.85 (1.01–3.37)*	1.01 (0.73–1.38)	1.06 (0.75–1.48)	1.18 (0.82–1.70)	0.96 (0.65–1.43)	0.84 (0.55–1.29)	0.77 (0.38–1.55)
Low	1.02 (0.61–1.69)	1.92 (1.09–3.38)*	0.88 (0.30–2.58)	0.68 (0.41–1.14)	1.03 (0.58–1.83)	1.14 (0.64–2.04)	0.92 (0.50–1.69)	0.63 (0.32–1.23)	0.48 (0.14–1.62)

Asterisks after the odds ratio (OR) of reference category correspond to the *P*-value for the improvement of the fit of the model by adding that variable to the model: *P < 0.05; **P < 0.01; ***P < 0.001.

SE differences were found in nutritional habits (skipping breakfast, daily consumption of fruit [among girls], daily concumption of vegetables [among girls], daily consumption of sweets [among girls]), physical inactivity, and smoking (among boys), but not in drunkenness, alcohol consumption, or cannabis use (Table 2). For skipping breakfast and physical inactivity, the SE gradient was similar for boys and girls (Table 3). As compared with the highest SE group, the lowest SE group had significantly higher proportions of breakfast-skippers and physically inactive adolescents, with ORs of approximately 1.5 and 1.6, respectively. However, the SE gradients of some other nutritional behaviors differed significantly (*P* < 0.05) by gender. While no SE differences were found in daily fruit or vegetable consumption among boys, girls from lower SE groups tended to behave less healthily (Table 2). For daily fruit consumption, a gender effect on SE differences was found for both the middle (OR, 1.51; CI, 1.09–2.10) and lowest SE groups (OR, 1.92; CI, 1.09–3.38), while for daily vegetable consumption there was a gender effect only for the middle group (OR, 1.85; CI, 1.01–3.37; Table 3). Boys from the lowest SE group had a significantly higher odds of eating sweets daily, as compared with their high SE group counterparts (OR 1.58). The only significant difference in the prevalence rates for substance use was for smoking among girls from the middle SE group who, after age adjustment, had a 1.38 times higher odds of smoking in comparison with their peers from the highest SE group. In all other groups, no significant SE differences in prevalence rates of substance use were found (Table 2). The gender effect on SE differences was not significant for any type of substance use (Table 3).

IDs were generally small, and the highest indices were for cannabis use among boys (6.48%) and smoking among girls (5.04%). In all other groups, the indices were lower than 4% (Table 2).

## DISCUSSION

Our study explored SE differences in several different HRB categories and the potential modification of these differences by gender. The results confirm that SE differences are generally small during adolescence. In addition, our findings show that SE gradients in adolescent HRB differ by gender only for some behaviors. Although more differences were found among girls than among boys in fruit consumption and vegetable consumption, the sizes of SE differences were similar between genders for other behaviors.

Our finding in a Central European country of only small SE differences in health behaviors with largely varying prevalence rates confirms previous evidence from Western Europe, which showed SE differences to be small or absent during adolescence.^6^^,^^35^ The largest SE inequalities were in nutritional behavior among girls. For physical inactivity, a slight SE difference was found between the highest and lowest SE groups in both genders. For substance use, almost no differences were found. This supports the theory of Richter,^3^ namely that parental SE position has little effect on adolescent substance use, as such behaviors have a later onset and are more influenced by peers than by family background. Although nutritional behavior and physical inactivity can be altered during adolescence, they stem from childhood, making them more subject to parental influence.^5^ The same process may also explain the relatively large SE difference in physical inactivity among both genders.

Interestingly, the overall prevalence rates of drunkenness were almost equal for boys and girls (19.6% vs 17.7%, respectively). However, for multiple alcohol use (≥3 times during the previous 4 weeks, without explicitly asking about drunkenness), the gap between boys and girls was much wider (17.5% vs 12.5%). This seems to contradict the claim of Richter^3^ that the trend toward gender-specific equalization in HRB applies more to patterns of moderate HRB, while male dominance remains for “harder” HRB. The results of our study show exactly the opposite, ie, minimal differences in the hard category of alcohol consumption and a higher prevalence in the more moderate category.

The strengths of this study are its large sample size and very high response rate. The study includes several HRB categories (nutritional behavior and physical inactivity, but also substance use), whereas other studies often focus on a single behavior.

A limitation of this study is the use of self-reports for data collection, which could have caused bias due to underreporting of risk behaviors.^36^ Underreporting may be more likely for use of substances that are either illicit (cannabis) or illicit in Slovakia for youths younger than 18 years (tobacco and alcohol). However, self-report was shown to be moderately valid, at least regarding alcohol use.^37^^,^^38^ Moreover, the present questionnaires were completed voluntarily and anonymously, which makes reporting bias less likely.^37^ Another limitation of this study is its cross-sectional design, which does not allow us to track possible alterations in SE gradient from childhood and adolescence until adulthood. Therefore, we cannot determine from our results that SE differences in Slovakia are less pronounced only in adolescence or how this relationship may change with age. Moreover, parental education is only 1 available measure of SEP. If a different measure of SEP (such as family income, parental occupations, or household assets) or a combination of such measures were used, the SE gradient for these behaviors could have differed as well. However, parental educational level is frequently used to assess the SEP of young people. Third, information on educational level of the parents was obtained from the adolescents, who might not know the education level of their parents. However, among those who returned the questionnaires, the response rate for these items was 96.9% and 94.1% for maternal and paternal education, respectively. Nevertheless, the adolescents may not have had accurate knowledge of parental education level.^39^ However, according to Lien et al,^40^ the strength of agreement between adolescent and parental reports of parental education was fair (κ coefficients, 0.30 and 0.38 for paternal and maternal education, respectively).

This study revealed that parental background, although not a very strong factor during adolescence, is related to some dietary habits, especially among females. It would be useful to explore the pathways of this relationship—for example, if their nutritional behavior is caused by direct intervention, example, advice, or global health consciousness of parents, or if parental SEP perhaps plays an indirect role by choosing peers with similar habits, as is often the case in substance use.^11^^,^^41^ In comparing present and past results, it is important to be aware that some studies used as a proxy of family SEP the highest educational degree achieved by each parent individually rather than the highest educational degree achieved by both parents. Finally, despite its relatively large sample, our study still had rather low statistical power to detect gender differences in the gradients for some behaviors, which indicates that the likelihood of false-negative findings is relatively large in those instances. However, this limitation relates only to relatively small differences that will generally be of less practical importance.

A very high proportion of students of both genders and in all SE groups did not eat vegetables daily. Thus, all adolescents should be targeted by prevention activities that aim to increase regular vegetable consumption. However, girls from middle and low SE groups, and boys from the lowest SE group, require special attention because the prevalence rates of other unhealthy nutritional behaviors were high in these groups, as was the prevalence rate of physical inactivity. The results suggest that, among girls, parents seem to have a more important role in consumption of fruit and a regular breakfast than in HRBs, which suggests that parents should be included in the promotion of these healthy nutritional habits.
